# Improved Draft Genome Sequence of *Clostridium pasteurianum* Strain ATCC 6013 (DSM 525) Using a Hybrid Next-Generation Sequencing Approach

**DOI:** 10.1128/genomeA.00790-14

**Published:** 2014-08-07

**Authors:** Michael E. Pyne, Sagar Utturkar, Steven D. Brown, Murray Moo-Young, Duane A. Chung, C. Perry Chou

**Affiliations:** a Department of Chemical Engineering, University of Waterloo, Waterloo, Ontario, Canada; b Graduate School of Genome Science and Technology, University of Tennessee, Knoxville, Tennessee, USA; c Biosciences Division, Oak Ridge National Laboratory, Oak Ridge, Tennessee, USA; d Neemo Inc., Hamilton, Ontario, Canada

## Abstract

We present an improved draft genome sequence for *Clostridium pasteurianum* strain ATCC 6013 (DSM 525), the type strain of the species and an important solventogenic bacterium with industrial potential. Availability of a near-complete genome sequence will enable strain engineering of this promising bacterium.

## GENOME ANNOUNCEMENT

*Clostridium pasteurianum* is a mesophilic, anaerobic Gram-positive bacterium that is apathogenic, can be easily cultivated in chemically defined media, and is more aerotolerant than many clostridia (1, 2). Historically, *C. pasteurianum* has been utilized extensively as a model organism for the study of nitrogen fixation (3) and clostridial ferredoxins (4). More recently, *C. pasteurianum* has received significant biotechnological attention because of its capacity to ferment waste glycerol (5) and glycerol-rich thin stillage (6), which are major by-products of biodiesel and bioethanol production, respectively. In addition to acids and carbon dioxide, glycerol is converted to appreciable quantities of butanol, 1,3-propanediol, and hydrogen gas (7), which have industrial potential as chemicals or biofuels. To allow genetic manipulation of this organism, several recent studies have outlined the need for a genome sequence of *C. pasteurianum* (5, 8). A concurrent effort has reported a draft genome sequence for the type strain (9), while partial or full genome sequences are also available for two *C. pasteurianum* isolates (BC1 [http://www.ncbi.nlm.nih.gov/GenBank/] and NRRL B-598 [10]), further demonstrating the appeal of this species. Here we report an improved draft genome assembly for the type strain of *C. pasteurianum*, which was generated using a hybrid next-generation sequencing approach.

The genome of *C. pasteurianum* ATCC 6013 was sequenced using 454, Illumina MiSeq, and single-molecule real-time (SMRT) RS I and RS II sequencing platforms with sequence coverages of 20×, 335×, 80×, and 90×, respectively. *De novo* genome assembly of PacBio data was performed using HGAP.1 protocol from SMRT Analysis software version 2.1. The resulting draft genome sequence of *C. pasteurianum* ATCC 6013 comprises 4,420,124 bp and 12 contigs, an improvement on the 37 contigs reported previously (9). The N50 contig size was improved from 229 kb to 859 kb. Geneious software (Biomatters Ltd., Auckland, New Zealand) identified two supercontigs, with putative contig orderings of ctg10-ctg1C-ctg5-ctg3C-ctg9-ctg12-ctg4-ctg11 and ctg6-ctg7-ctg2-ctg8, respectively (C=complement of contig). Mapping of Illumina and 454 reads against a PacBio assembly detected only 5 SNPs, which were corrected and defines the high quality of assembly resulting from PacBio reads.

Genome annotation was performed using the Oak Ridge National Laboratory annotation pipeline, based on the Prodigal gene prediction algorithm (11). Ribosomal RNAs were annotated using RNAmmer 1.2 (12), and transfer RNAs were predicted using tRNAscan-SE (13). The G+C content of the genome is 30%. Approximately 4,047 protein-coding, 95 tRNA, and 29 rRNA (14 × 16 S and 15 × 23 S) genes were predicted in the genome. No putative extrachromosomal elements were identified. Gene sequences for enzymes involved in the production of primary metabolites, including ethanol, butanol, and 1,3-propanediol, are present in the genome sequence, many of which are organized in operons similar to *C. acetobutylicum* (14). An acetone formation locus (*adhE*-*ctfAB*-*adc*) possessing significant sequence similarity to that of *C. acetobutylicum* (15) is encoded in the genome, yet, as indicated previously (9), acetone production by *C. pasteurianum* has not been reported. It is expected that the draft genome sequence presented herein will guide future strain improvement efforts involving *C. pasteurianum* (16).

### Nucleotide sequence accession numbers.

This Whole Genome Shotgun project has been deposited at DDBJ/EMBL/GenBank under the accession JPGY00000000. The version described in this report is version JPGY01000000.
